# Supplementary material for: Evaluating access to oral anti-diabetic medicines: A cross-sectional survey of prices, availability and affordability in Shaanxi Province, Western China
Source: PLoS One. 2019 Oct 16;14(10):e0223769. doi: 10.1371/journal.pone.0223769 (PMC6795464; doi:10.1371/journal.pone.0223769)
Supplement: S1 File — (DOCX) [file pone.0223769.s001.docx]

Medicine Price and Availability Data Collection Form

Researcher： Date： Facilities：

| **Drug name** | **Dosage forms** | **Strength** | **Pack size** | **Price Per Pack** | **Price-Per-Unit** | **Remarks** |
| --- | --- | --- | --- | --- | --- | --- |
| Metformin | Bristol-Myers Squibb（Glucophage） | 500 mg |  |  |  |  |
|  | Bristol-Myers Squibb（Glucophage） | 800 mg |  |  |  |  |
|  | Tab | 250 mg |  |  |  |  |
|  | Tab | 500 mg |  |  |  |  |
|  | SR Cap | 250 mg |  |  |  |  |
|  | SR Tab | 500 mg |  |  |  |  |
|  | R Cap/Tab | 250 mg |  |  |  |  |
|  | R Cap/Tab | 500 mg |  |  |  |  |
| Glibenclamide | Tab | 2.5 mg |  |  |  |  |
| Gliclazide | Servier（Diamicron） | 30 mg |  |  |  |  |
|  | Servier（Diamicron） | 80 mg |  |  |  |  |
|  | SR Tab | 30 mg |  |  |  |  |
|  | Tab | 80 mg |  |  |  |  |
| Glipizide | Pfizer（Glucotrol XL） | 5 mg |  |  |  |  |
|  | Tab | 2.5mg |  |  |  |  |
|  | Tab | 5 mg |  |  |  |  |
|  | Cap | 5 mg |  |  |  |  |
| Glimepiride | Sanofi（Amaryl） | 2 mg |  |  |  |  |
|  | Tab | 1 mg |  |  |  |  |
|  | Tab/Cap | 2 mg |  |  |  |  |
| Acarbose | Bayer（Glucobay） | 50 mg |  |  |  |  |
|  | Tab/Cap | 50 mg |  |  |  |  |
| Repaglinide | Novo Nordisk（NovoNorm） | 1 mg |  |  |  |  |
|  | Novo Nordisk（NovoNorm） | 2 mg |  |  |  |  |
|  | Tab | 0.5 mg |  |  |  |  |
|  | Tab | 1 mg |  |  |  |  |
|  | Tab | 2 mg |  |  |  |  |
| Pioglitazone | Takeda（Actos） | 15 mg |  |  |  |  |
|  | Tab | 5 mg |  |  |  |  |
|  | Tab/Cap | 15 mg |  |  |  |  |
|  | Tab | 30 mg |  |  |  |  |
